# The Effectiveness of Psychoeducational and Financial Intervention to Support Caregivers of Individuals With Alzheimer’s Disease in Poland

**DOI:** 10.1093/geroni/igz026

**Published:** 2019-08-02

**Authors:** Magdalena Leszko

**Affiliations:** Institute of Psychology, University of Szczecin, Poland

**Keywords:** Alzheimer’s disease, Dementia, Caregiving, Caregiver burden, Interventions

## Abstract

**Background and Objectives:**

The goal of this study is to create data-driven guidelines and tools for caregivers and health care professionals that will enable caregivers to be prepared for future caregiving-related challenges and decrease their levels of stress.

**Research Design and Methods:**

A total of 60 spousal caregivers of individuals with Alzheimer’s disease (AD) living in Poland were recruited for this study. The participants were assigned to two different groups. The intervention group was provided with a five-session training focused on teaching coping strategies for managing difficult behaviors and provided with a stipend for a period of 6 months (a financial aid program recently launched by the local agency). A control group was not provided with any of the interventions but asked to complete the questionnaires. The effectiveness of each intervention was assessed at a baseline and 6 months after baseline evaluation.

**Results:**

Caregivers who received financial and educational training reported significantly decreased levels of depression and caregiver burden in comparison to the control group. The majority of caregivers emphasized that caregiving created financial problems and that their work has been underestimated by the government.

**Discussion and Implications:**

This project addresses several issues of central importance to the success of building research foundation for the interventions. The results have the potential of generating more efficient and personalized interventions that address the needs of the caregiver as they occur, leading to increased knowledge of AD and decreased levels of depression and caregiver burden.

**Translational Significance**

The findings of this research demonstrated that combing financial and psychoeducational interventions are effective in reducing caregiver burden and depression among spousal caregivers of individual with Alzheimer’s disease. The findings also underscore the importance of providing educational materials that can improve caregivers’ knowledge about the disease.

Research in the area of caregiving, especially of adults with Alzheimer’s disease (AD), is of increasing importance at this time. AD is a progressive neurodegenerative disorder that significantly impairs a person’s quality of life (Justice, 2018). It is estimated that there are approximately 50 million individuals worldwide living with AD or a related form of dementia (Alzheimer’s Association, 2018). Although the exact number of individuals suffering from AD in Poland in unknown, recent estimates suggest that nearly half a million Polish people (7.9% of all individuals age 65 and older) can be affected by dementia. This number is projected to quadruple by 2050 (The Supreme Audit Office [NIK], 2017). Increasing age is the greatest risk factor for AD; thus, the number of individuals affected by this disease will grow as the population continues to grow. It is therefore highly likely that many older couples will face this disease at some point in their lives.

Caregivers often report feeling unprepared and having inadequate knowledge and skills to provide care (Reinhard, Given, Petlick, & Bemis, 2008). Some of the most common behavioral symptoms of AD such as aggression and wandering can be challenging and stressful for caregivers because they do not know how to respond to difficult behaviors (Feast et al., 2016; Gitlin, Kales, Marx, Stanislawski, & Lyketsos, 2017). As a result of stress due to the unpredictable nature of the symptoms and increasing caregiving demands, caregivers may experience serious negative health consequences, such as caregiver burden, depression, social isolation, or even premature mortality (e.g., Bevans & Sternberg, 2012; Sheung-Tak, 2017). In addition, caregivers often express feeling angry, drained, frustrated, guilty, or helpless (Center on an Aging Society, 2005). Previous studies demonstrated that strengthening caregivers’ competence and confidence in providing care and knowledge about the disease may result in experiencing higher levels of mastery and decreasing levels of burden (Cameron, Herridge, Tansey, McAndrews, & Cheung, 2006; Van Den Wijngaart, Vernooij-Dassen, & Felling, 2007). Taking into account that the number of people affected by AD is increasing at an alarming rate and that the majority of these individuals lives at home and receives care from their family members (Lepore, Ferrell, & Wiener, 2017; Schulz & Martire, 2004), it is not surprising that much effort has been expended on developing nonpharmacological interventions that could protect caregivers from experiencing excessive burden by improving caregivers’ knowledge, confidence, and abilities to effectively react to dementia-related symptoms.

Although there is no agreed on categorization among researchers, nonpharmacological caregiver interventions are typically broken into six types: (a) psychoeducational interventions, (b) supportive interventions, (c) respite/adult day care, (d) psychotherapy, (e) interventions to improve care receiver competence (behavior management/skills training), and (f) multicomponent interventions (combinations of, e.g., educational interventions, support, psychotherapy, and respite; Sörensen, Pinquart, & Duberstein, 2002). The majority of research focused on the effectiveness of psychoeducational interventions that typically aim at improving caregiver’s well-being and skills by providing information about the disease, its process, and resources that a caregiver may use (Pinquart & Sörensen, 2003). This type of intervention underscores the importance of training caregivers to respond effectively to disease-related problems and has consistently been found to be effective in improving subjective well-being and knowledge about the disease while decreasing depressive mood among caregivers of individuals with AD (e.g., Brodaty & Arasaratnam, 2012; Gitlin, Hodgson, Jutkowitz, & Pizzi, 2010; Hepburn, Lewis, Sherman, & Tornatore, 2003).

Although providing psychoeducational intervention is considered an important strategy for improving quality of care and caregiver’s well-being and fostering problem-solving skills, the findings regarding decreasing the levels of caregiver burden are mixed. A number of studies have found a significant decrease in caregiver burden, whereas other studies found that although interventions helped to improve knowledge, they did not reduce the burden (Brodaty, Green, & Koschera, 2003; Selwood, Johnston, Katona, Lyketsos, & Livingston, 2007; Sörensen et al., 2002). Some researchers concluded that implemented alone, psychoeducational interventions may not be a sufficient way of reducing the burden (Griffin et al., 2015; Whittier, Coon, & Aaker, 2002).

There are many elements contributing to the high levels of caregiver burden. Because of its objective and subjective nature, the caregiver burden is a multidimensional experience (Carretero, Garces, Rodenas, & Sanjose, 2009). Although teaching caregivers how to manage specific care-recipient’s behavioral problems can improve the caregiver’s well-being, reduce the fear of having inadequate knowledge, and help caregivers to become more confident in providing care, research shows that the financial costs associated with family caregiving are also a significant factor in predicting caregiving burden (Lai, 2012). There are studies looking at the monetary burden to society and the country’s economy (e.g., Hurd, Martorell, Delavande, Mullen, & Langa, 2013; Morris et al., 2015), but little is known about the financial challenges experienced by the individual, informal caregivers. Many families not only cannot afford the cost of institutional care but also struggle financially to cover home-based services (Bookman & Kimbrel, 2011). According to the survey conducted by the National Study of Caregiving (NSOC), one third (31.3%) of the caregivers reported having financial difficulties related to caregiving (Kasper, Freedman, & Spillman, 2013). Out-of-pocket expenses play a significant role in providing care and are mostly used to pay for medical services, food, travel costs, clothing, medical equipment, and legal fees (National Academies of Sciences, Engineering, and Medicine, 2016). Although the data on out-of-pocket costs are limited, a survey undertaken by Evercare and NAC in 2007 indicated that caregivers spent an average annual amount of USD 5,531. Not only the financial hardship influences the caregiver’s current situation and may lead to taking on debt but may also have long-term effects. The financial strain due to caregiving can be especially detrimental for women as they are the majority of caregivers (National Alliance for Caregiving and AARP, 2015). For example, Wakabayashi and Donato (2006) found that caregiving increases the likelihood that women experience poverty and reliance on public assistance. Although researchers emphasize the role of financial strain as one of the predictors of caregiver burden (Cheng, 2017; Schulz & Eden, 2016), no one, to the best of our knowledge, conducted an intervention study in which caregivers would receive monetary support.

Considering that there is no treatment that could cure AD or stop its progress, developing effective and low-cost nonpharmaceutical interventions with the aim to improve and support the quality of life in care recipients and their caregivers has become a major public health concern (World Health Organization, 2018). The 

purpose of this article is to extend the line of research on caregiver interventions by testing whether a combination of psychoeducational and financial intervention will reduce depression and caregiver burden among spousal caregivers of individuals suffering from AD who live in Poland.

Similar to other developed countries, Poland’s population is aging. In 2016, the number of individuals aged 65 and older constituted 16.3% of the total population and is projected to increase to 24.5% by 2035 (European Commission, 2018). The growing number of individuals with AD may pose challenges for families and the health care system. Although Poland provides public health care to all of its citizens and covers a vast range of health care services, almost half of Polish residents (47%) reported using private health care because of the growing dissatisfaction with the waiting times for outpatients services (Centre for Public Opinion Research, 2016). Because many services, medical devices, and medications are not covered by the National Health Fund, costs associated with paying for them may be a financial burden for those with chronic health conditions. The majority of older adults reported that their household expenditures on medical services are high and sometimes they could not afford to buy prescribed drugs (Statistics Poland, 2019).

The majority of individuals with AD in Poland (92%) are cared for by family members (Durda, 2010). This might be due to culturally strong family ties (Golinowska, 2010) or high costs of nursing homes. The nursing homes boarding costs are set at 250% of the minimum old-age pension or 70% of the resident’s monthly income (whichever is lower; Sagan et al., 2011). Unfortunately, financial assistance for family caregivers is very limited. Caregivers are entitled to apply for a nursing allowance of PLN 620 (USD 162) per month if they have given up jobs to care for family members. However, the allowance is granted only if the net aggregate income of the caregivers is lower than PLN 764 (USD 200) per person per month (The Polish Social Insurance Institution, 2018). In comparison, the average income before tax in Poland is PLN 4,900 a month (PLN 3,530 after tax, USD 927; Statistics Poland, 2018).

To date, no caregiver interventions have been conducted in Poland. This research aims to broaden the current literature on interventions by testing the effectiveness of psychoeducational and financial interventions among spousal caregivers of individuals suffering from AD. Previous studies suggested that psychoeducational intervention implemented alone may not be as effective as a multicomponent intervention (e.g., Gallagher-Thompson & Coon, 2007; Whittier et al., 2002). Therefore, based on the literature, the authors believed that combining psychoeducational and financial interventions would yield a positive effect and result in decreased levels of caregiver burden and depression. The study focuses on spousal caregivers because they face greater challenges of providing care than an adult child (Adelman, Tmanova, Delgado, Dion, & Lachs, 2014). Compared with other caregivers, spouses spend more time providing care (Keating & Fast, 1999). Spousal caregivers of individuals with AD may experience increased burden not only because the demanding and exhausting tasks (e.g. lifting a care recipient or assisting with basic needs) accumulate as the disease progresses, but also because of their age-associated health problems. Several studies revealed that one half of caregivers suffer from at least one chronic health condition themselves (Collins & Swartz, 2011; Schulz, O’Brien, Bookwala, & Fleissner, 1995) and caregiver’s own health problems are often reported to interfere with the provision of care (Connell & Gibson, 1997). Of those caring for someone aged 65 and older, the average age is 63 years old with one third of these caregivers in fair to poor health (Family Caregiver Alliance, 2019).

To determine the effects of the intervention, we compared the results to individuals assigned to the control group which did not receive the intervention. In addition, because certain aspects of caregiving can be easily overlooked when researcher focuses only on quantitative data, in this study we collected both qualitative and quantitative data.

## Research Design and Methods

### Procedure

The study was conducted in Szczecin, Poland. Eligible participants were primary caregivers for a spouse with AD, living with their spouse at home, and with intact abilities of daily living and no diagnosis of dementia. Caregivers who were caring for bed-bound individuals we excluded because being bed-bound is a risk factor for institutional placement or death (Belle et al., 2006) and we wanted to ensure that the caregiver will be still providing care during the 6-month follow-up interview.

The research team obtained a list of individuals who were eligible to receive the financial aid and agreed to be contacted by the research team. To be eligible to receive the financial help, care recipients had physician-diagnosed (by a neurologist or psychiatrist) AD and were at least 75 years old. Prior to recruitment, letters describing the study were sent out to caregivers inviting their participation. Of 124 potential participants who fulfilled eligibility criteria, 33 (27%) agreed to participate in the study. Caregivers who were not eligible for financial aid were invited to participate in the study by professionally designed brochures describing the project goals, which were distributed in primary care clinics, social service agencies, physician offices, and churches. The research team also posted announcement of the study online, resulting in telephone calls from 42 eligible caregivers; 30 entered into the study. The study was approved by a University-based Intuitional Review Board. All caregivers who participated in the study provided written informed consent. Upon providing the consent, a semistructured interview was used to elicit data. All interviews were conducted face-to-face in caregivers’ houses. Participants who received financial aid were assigned to the intervention group and participants who were not eligible for financial aid were assigned to the control group.

The intervention group was provided with a five-session psychological training focused on teaching coping strategies in managing difficult behaviors conducted by interviewers with extensive experience in psychology and gerontology. During these five sessions, caregivers of individuals with AD had an opportunity to express their worries and discussed plan for the future as the disease progresses. During the sessions, caregivers were given practical tips on how to prevent or deal with certain symptoms of the disease such as difficulties communicating, handling crisis situations, mood swings, wandering, and delusions. Throughout the program, caregivers learned about available resources and helpful websites, and were provided with educational materials about dementia. The psychoeducational intervention was delivered individually during one-to-one sessions. The intervention group was also provided with a stipend for a period of 6 months. This financial aid program was launched by the local government. The participants received PLN 1,000 (USD 278) every 3 months. As of 2018, the national average pension in Poland is PLN 2,180 (USD 603).

A control group was not provided with any of the interventions, but the participants were interviewed and asked to complete the questionnaires. We evaluated the effectiveness of each intervention at approximately 6 months from baseline. The follow-up visit focused on the effectiveness and satisfaction with the intervention. Each participant received USD 20 gift card as compensation for participation in this research.

#### Sample

Initially, the two recruitment sources yielded a total of 63 spousal older caregivers who entered into the study. Shortly after the baseline interview, one care recipient died, and two caregivers could not participate because of hospitalization. The final sample comprised 60 caregivers between the ages of 65–88 years (*M* = 76.2, *SD* = 5.53). There were 42 women and 18 men in the sample. All participants were white, native speakers of Polish, what reflects the composition of the geographical area. Their educational level was generally high—the majority of participants (23) from the intervention group (23) and from the control group (22) obtained higher than high school diploma education. Only three participants from the intervention group and three from the control group reported receiving a high monthly household income. The remaining participants were in either low-income or middle-income brackets.

#### Measures

Participants were asked to complete a set of questionnaires that inquired about their sociodemographic variables (age, highest level of education, income level) and measured their levels of depression, caregiver burden, and knowledge of AD. The qualitative component of this study aimed at gaining a better understanding of caregivers’ needs and their opinions about the intervention. The semistructured interviews covered several topics including the experience of receiving the diagnosis, experiences providing care, satisfaction with available health care services, current problems with symptoms management, caregivers concerns about providing care, and the quality of the relationship between the caregiver and his or her spouse. Interviews were transcribed verbatim and subjected to coding and content analysis. The interviews took on average 90 min, were digitally recorded, and were transcribed verbatim. The same questionnaires were given prior and post-intervention.

Because caregiver burden and depressive symptoms are often the most common negative outcomes of providing care for the older adults (Jepson, McCorkle, Adler, Nuamah, & Lusk, 1999; Schulz & Sherwood, 2008), we focused on these variables. Surveys were completed without any missing data in the variables included in the study.

##### Depressive symptoms

Depressive symptoms were measured with the Polish version of the Beck Depression Inventory (BDI), a 21-item self-report measure using a 4-point scale indicating degree of severity; items are rated from 0 (not at all) to 3 (extreme form of each symptom). The statements express feelings common in depression (e.g., sense of failure, suicidal ideation, guilt, low self-worth, and social withdrawal). The score is calculated by adding the rating for the 21 items, with a maximum total score of 63. Higher scores indicate greater depressive severity (Beck, Brown, Steer, Eidelson, & Riskind, 1987). The psychometric analyses of the Polish translation indicated very high reliability and validity, are fully equivalent to the original version, and proved to be a very useful tool for use in scientific research and clinical practice to measure depression among Polish-speaking participants (Zawadzki, Popiel, & Praglowska, 2009).

##### Caregiver burden

Caregiver burden was assessed using the Burden Scale for Family Caregivers (BSFC). The BSFC is a 28-item instrument for measuring subjective burden in informal caregivers. Each item is a statement that is rated on a 4-point scale with the values “strongly disagree” (0), “disagree” (1), “agree” (2), and “strongly agree” (3). A high degree of agreement indicates higher subjective burden for the caregiver (Gräßel & Leutbecher, 1993). The scale is designed for use in both clinical practice and research studies and is available for free in 20 languages what allows for the comparison of international research findings.

##### Knowledge about Alzheimer’s disease

Knowledge about AD was assessed using the Alzheimer’s Disease Knowledge Scale (ADKS), a 30-item, true/false scale that comprises statements about risk factors, assessment and diagnosis, symptoms, course, life impact, caregiving, and treatment and management of AD. A total score is calculated by summing the number of correct responses (Carpenter, Balsis, Otilingam, Hanson, & Gatz, 2009).

##### Self-rated health

Self-rated physical and mental health were assessed with a single item, “Overall, how would you rate your physical and mental health?”. The respondents rated their own physical and mental health as (1) very good, (2) good, (3) fair, (4) poor, or (5) very poor. Scores on the Self-rated Health scale ranged from 0 to 5, with higher scores indicating worse mental and physical health.

#### Demographics

Sociodemographic and health variables included caregiver’s age, gender, educational attainment, and amount of time spent daily on providing care. Age was coded in years, and gender was dichotomized with 1 indicating female. We used three categories of educational attachment: high school or less, some college, and college degree or higher. Caregiver’s household income was divided into three groups: low (less than 2,000 PLN), middle (2,000–3,000 PLN), and high (more than 3,000 PLN).

#### Data analysis

Descriptive statistics were calculated for all variables. We compared the two groups using chi-square test across categorical measures and independent *t*-test across continuous variables. *T*-tests were used to compare baseline and post-intervention follow-up scores. All analyses were developed using SPSS. The qualitative method of framework analysis was employed to identify core concepts emerging from the interviews (Gale, Heath, Cameron, Rashid, & Redwood, 2013). Transcripts were analyzed by two principal investigators of the study.

## Results

### Descriptive Data

Table 1 presents the descriptive statistics of caregivers from the intervention and control groups. The average level of caregivers’ depression was 14.3 (*SD* = 6.9) in the intervention group and 13.8 (*SD* = 6.2) in the control group. The mean score of caregiver burden was 41.1 (*SD* = 8.8) in the intervention group and 37.6 (*SD* = 9.2) in the control group. Regarding the knowledge of the AD, participants in the intervention group scored on average 21.1 (*SD* = 2.7) points, whereas the average score of participants in the control group was 37.6 (*SD* = 9.2).

**Table 1.** Baseline Sample Characteristics

Variables	Financial and psychological intervention (*n* = 30)	Control group (*n* = 30)
	Mean (*SD*)	Mean (*SD*)
Age (years)	78.3 (6.3)	74.1 (3.7)
Female (%)	73.3	66.7
Education, *n* (%)		
High school diploma or less	7 (22.3)	8 (25.8)
Some college but no degree	14 (46.7)	14 (45.2)
College degree and higher	9 (30)	8 (25.8)
Income, *n* (%)		
Low	17 (56.7)	14 (46.7)
Middle	10 (33.3)	13 (43.3)
High	3 (10.0)	3 (10.0)
Time since diagnosis in years	5.3 (2.1)	4.7 (1.6)
Self-report of health	3.28 (0.92)	3.3 (0.95)
ADKS	21.1 (2.7)	20.6 (2.7)
BDI	14.3 (6.9)	13.8 (6.2)
BSFC	41.1 (8.8)	37.6 (9.2)

*Notes*: ADKS = Alzheimer’s Disease Knowledge Scale; BDI = Beck Depression Inventory; BSFC = Burden Scale for Family Caregivers.

### Baseline Measures

The intervention and control groups were similar across outcome measures at the baseline. As given in Table 1, the average age of caregivers in intervention sample was 78.3 years, and the average time of proving care in years was 5.5 years. Wives accounted for 73.3% of the sample. The mean age of caregivers in the control group was 74.1 (*SD* = 3.7), and the average time of providing care was 4.7 years (*SD* = 1.6). Caregivers in the intervention group were significantly older in comparison to the control group, *F* (2, 58) = 17.1, *p* = .000. No significant differences were found between the two groups in the BDI, ADKS, BSFC, and self-rated health.

We also conducted correlational analyses. As given in Table 2, depression was significantly positively correlated with caregiver burden (*r* = .26, *p* < .05) and self-rated health (*r* = .37, *p* < .05) only among caregivers in the intervention group.

**Table 2.** Correlation Matrix of All Study Variables

	1.	2.	3.	4.	5.	6.	7.	8.	9.
1. Age		−.06	−.46**	−.13	.29*	−.06	−.10	−.09	−.01
2. Gender	−.05		−.03	−.50**	−.31	.06	−.16	.08	−.11
3. Education	−.18	−.10		.33	.22	−.13	.13	−.02	.08
4. Income	−.12	−.20	.41*		.40*	.01	−.05	.04	.11
5. Time since diagnosis	.31	.12	.00	.20		.17	.18	−.18	.27
6. Depression	−.20	.08	−.29	−.25	.00		.14	.26*	.37*
7. ADKS	−.10	.15	−.09	−.08	.02	.17		.05	.10
8. Burden	−.02	.48**	−.24	−.19	.08	.31	.22		−.09
9. Self-rated health	.07	.13	.24	−.04	0.30	.18	.33	−.04	

*Notes:* ADKS = Alzheimer’s Disease Knowledge Scale; Depression = Beck Depression Inventory; Burden = Burden Scale for Family Caregivers. Correlations below the diagonal are for the caregivers in the control group. Correlations above the diagonal are for the caregivers in the intervention group.

*p < .05 level (two tailed); **p < .01 level (two tailed).

### Follow-up

There was a significant difference in the scores measuring caregiver burden among caregivers in the intervention group. Caregiver burden and depression declined after the intervention, *t*(29) = 4.56, *p* < .001, *d* = .83; *t*(29) = 2.23, *p* = .03, *d* = .40, respectively (see Table 3). At 6-month follow-up, the intervention group showed a significant increase in scores measuring AD knowledge, *t*(29) = −4.69, *p* < .001, *d* = .86. No significant differences were found in self-reported health. There were no statistically significant differences over the 6-month study period in the control group, although the results showed a nonsignificant increase in the scores in regard to caregiver burden.

**Table 3.** Comparison Between Baseline and Follow-up Outcomes

	Intervention group				Control group			
Variable	Baseline	Follow-up	*p* Value	Cohen’s *d*	Baseline	Follow-up	*p* Value	Cohen’s *d*
	Mean (*SD*)	Mean (*SD*)			Mean (*SD*)	Mean (*SD*)		
BSFC	41.1 (8.8)	34.2 (10.6)	<.001	.83	37.6 (9.2)	38.21 (9.1)	.09	.32
BDI	14.28 (6.9)	10.9 (6.8)	.03	.40	13.8 (6.2)	14.0 (5.9)	.14	.28
ADKS	21.1 (2.7)	23.7 (2.4)	<.001	.86	20.6 (2.7)	20.8 (2.6)	.09	.32
Self-rated health	3.28 (0.92)	3.33 (8.4)	.69	.08	3.3 (0.95)	3.4 (0.8)	.31	.18

*Notes*: ADKS = Alzheimer’s Disease Knowledge Scale; BDI = Beck Depression Inventory; BSFC = Burden Scale for Family Caregivers.

### The Interviews

We present results of the qualitative part of our study. Themes that arose during the interviews included sources and barriers in obtaining information about the disease, the most overwhelming aspects of providing care and caregivers’ perspectives on what interventions and policies should be implemented to help caregivers with AD.

### Knowledge About Alzheimer’s Disease

Caregivers were asked about their sources of information about the AD.

The majority of participants reported receiving booklets from their doctors but at the same time emphasized that the booklets included only basic information.

They say that its important to meet and talk with other family members. Nobody has time for that. Talking wont help. I need information about what to do and expect.

Although some caregivers were willing to learn more about the disease, the materials they found while looking for reliable information were too difficult to understand because of the medical jargon. Caregivers also expressed lack of comprehensive materials that would cover managing the most common symptoms of the disease. Consequently, they often felt overwhelmed and did not know what to do.

Its difficult when I dont know what made him angry, why he gets so upset sometimes and it makes me angry. I feel frustrated.

A few participants stated that they never looked for information about the disease. One participant mentioned help in acquiring information from his adult child:

My daughter reads online and tells me what to do.

#### The most difficult aspects of providing care

When asked about the most difficult part of proving care, all participants mentioned problems with performing activities of daily living such as eating and personal hygiene, nail care, shaving, and dressing up. A few caregivers also talked about lifting the care recipient as one of the most difficult part of caring.

#### Satisfaction with the financial help

The majority of caregivers reported spending the financial aid on medications, diapers, and medical devices (e.g., special mattresses). Participants considered the financial help as being an important aspect of helping them to meet to current needs (e.g., buy medications) or plan for the future expenses.

Its a big help from the government. Im aware that with the progression of the disease I will need to hire somebody to help me and every cent counts.

A few participants mentioned that although financial aid is crucial, respite care would benefit them greatly.

I need someone who could take my husband for a few hours and bring him back. I know there are services like that abroad. My sons mother-in-law has dementia and she has two formal caregivers they are paid for by the government. She doesnt have to worry about anything because they are there around the clock.

## Discussion and Implications

With projected increase in the number of individuals with AD, caregivers will become even more essential for decreasing the government spending associated with escalating health care costs. Research shows that care provided by family members lowers hospital admission rates among older adults and is associated with a lower risk of nursing home admission (Schulz & Eden, 2016). Therefore, the availability of informal caregivers allows the government to reduce costs associated with health care system and long-term services. While informal caregivers are important for the health care system, researchers have long recognized that caregivers may report higher levels of depressive symptoms and impoverished physical health in comparison to noncaregivers. The detrimental effects of caregiving on physical and mental health stem from the primary stressors experienced by the caregivers, such as the duration and type of care provided, care recipient’s behavior problems, functional disabilities, and financial strain (Pinquart & Sörensen, 2003, Vitaliano, Zhang, & Scanlan, 2003). These factors may contribute to the experience of caregiver burden and poor mental health, which in turn may increase the risk of nursing home admission (Dunkin & Anderson-Hanley, 1998). Recognizing the importance of reducing caregiver burden, creating effective nonpharmacological intervention aiming at reducing the amount of stress experienced by the caregiver has become more critical for researchers, health care professionals, policy makers, and families of individuals with AD.

Although the interventions are beneficial for caregivers and may delay nursing home placement, previous studies have demonstrated small and inconsistent benefits of certain types of intervention in reducing the caregiver burden. For example, several psychoeducational interventions were found to reduce depression, but no effect was found on burden and role strain. Inconsistencies across the studies may have been due to a variety of factors including methodological differences. Addressing this issue, several researchers suggested that using one type of intervention might not be as effective as applying a multicomponent intervention because they address a wider range of caregiver’s health outcomes (Griffin et al., 2015; Whittier et al., 2002). Therefore, in this study we test the effectiveness of the combined psychoeducational and financial intervention on caregiver’s depression, caregiver burden, and knowledge of the AD. The secondary aim of this study was to build the clinical groundwork for the low-cost interventions for individuals with AD and their caregivers and promote long-term research that contributes to caregivers’ improved health outcomes and decreased rates of institutionalization. We collected data from 60 caregivers during face-to face interviews. Thirty participants took part in an intervention during which they received financial aid and psychological sessions increasing their knowledge of AD. The other 30 participants were in the control group. The effectiveness of the intervention was measured by changes in scores related to depression, caregiver burden, and knowledge of AD prior to the intervention and at the 6-month follow-up.

Our findings confirm previous studies indicating that caregivers who experience higher levels of depression report greater caregiver burden and worse self-rated health (e.g., Kim, Chang, Rose, & Kim, 2012; Sherwood, Given, Given, & von Eye, 2005). The results of this study showed that the implementation of both, financial and psychological interventions, significantly improved caregivers’ knowledge of AD and reduced depression and caregiver burden in comparison to the control group. The qualitative aspect of our study demonstrated that caregivers receive insufficient information from their health care professionals. They often feel overwhelmed and do not know what to do when facing challenging situations. Participants in the study stated that knowledge gained from helped them to manage challenging behaviors such as aggression or problems with bathing. The caregivers also added that now they understand the symptoms better and realized that their spouse out-of-character mood changes are result of the disease rather than deliberate behavior.

Financial burden is a common factor associated with caregiver burden. The out-of-pocket caregiving expenses reported by the participants included a wide range of spending, such as transportation, food, medications, equipment (e.g., special mattresses or beds), and home modifications (e.g., bathroom remodeling) to ensure safety and make it easier to navigate around the house. The majority of participants was not able to give the exact estimates of the caregiving costs but emphasized that they were significant. Thus, state financial support programs play a crucial role in decreasing caregiver burden. The financial help from the government was considered as a good idea as caregivers could pay for medications or save money to hire a formal caregiver. Future work should consider collecting more precise data on caregivers’ expenses to improve our understanding of the economic effects of caregiving.

In this study, we combined quantitative and qualitative approach. The open-ended questions allowed participants to answer the questions using their own words, rather than choosing from one of the preformulated responses. Letting the participants answer in their own words about the effectiveness of intervention brought some unique responses, which led to findings that might otherwise have never emerged if the responder only selected one of the predefined answers. Thanks to this mixed-methods design, we learned that the lower scores in the AD knowledge scale can be partially attributed to the obstacles in obtaining the information. Participants may not have knowledge about the disease and its symptoms management because they do not receive enough information from their health care professionals or the available materials are written using complicated and difficult to understand language. Studies conducted in general population have demonstrated that patients do not always understand prescription instructions and often forget a significant amount of information they received from their health care professionals (Jimmy & Jose, 2011). Thus, providing caregivers with reliable materials, written in everyday language may increase their understanding of the disease process. We also learned that it is important to examine specific components of burden instead of relying on the global scores. In this study, the majority of participants indicated that the behavioral symptoms and helping with activities of daily living were the most challenging for them. Although self-efficacy in symptoms management contributes to caregiver burden, studies indicate that loneliness and social isolation are also significant factors. Therefore, future studies should examine how the impact of particular components of caregiver burden changes as the disease progresses.

The quantitative finding shed light on our understanding of the effect of caregiving and effectiveness of the intervention. One important finding that emerged from the interviews was that caregivers often felt abandoned by the government and left alone by the rest of society. Thanks to the financial support, they were able to purchase necessary out-of-pocket expenses (e.g., drugs, clothes, transportation) and felt more appreciated for the work they do. Some caregivers were also able to hire an informal caregiver for respite care which brought a temporary relief from everyday chores.

Caregiving has become an important issue in an aging society. Caring for a spouse may be a stressful experience (Pinquart & Sörensen, 2003; Schulz & Beach, 1999) that leads not only to negative health outcomes but also to financial strain. Our findings extend prior work by examining the role of multicomponent intervention that combines financial aid and psychoeducational intervention. Financial aid provided by the government help caregivers to buy medication and medical devices, whereas the educational materials and conversations with a psychologist provided the caregivers with support and help understand some misconceptions about the disease and improved caregivers’ knowledge by teaching them practical solutions that may be needed to provide the best possible care.

Several limitations of the study should be noted. First, the findings from this study are limited because the data were collected in Poland and included a small sample from the same geographical area what may decrease the generalizability of the study. The second limitation is that our approach was to combine both types of interventions; therefore, we do not know which had the strongest effect. To overcome this limitation, future studies should employ groups in which one receives only psychoeducational training, one receives financial aid, and the third one receives a combination of both interventions. We also assessed depression, caregiver burden, and knowledge of the AD only two times: at the baseline and at the follow-up assessment. The scales refer to the symptoms experienced in the last month and did not allow us to see the changes between the baseline and the follow-up assessment. Future research should assess the symptom of depression and caregiver burden more frequently to identify changes in caregiver’s outcomes. The next limitation lies in the sample, which included only married couples. We focused on spousal caregivers as they are a unique group of family caregivers. They are more susceptible to feelings of stress than other caregivers (Zarit, Davey, Edwards, Femia, & Jarrott, 1996) and they may have fewer resources than other caregivers (Seltzer & Li, 2000). However, relying only on spousal caregiver limits the generalizability of the findings. The participants in our intervention group were significantly older than spousal caregivers in the control group. Future research should employ participants with similar demographic characteristics.

Despite the limitations, results from this study offered important insights into the caregiving experience for both psychologist and other health care professionals because they also play an important role in decreasing the rate of burden. The majority of caregivers reported obtaining knowledge about the disease form their physicians but emphasized that they were unaware of other available sources. Because health care professionals are the primary source of reliable information for many caregivers, they should be able to identify caregiver knowledge gaps and guide them to specific sources of information, including printed materials, websites, and referrals to other health care professionals (Peterson, Hahn, Lee, Madison, & Atri, 2016). By guiding the caregiver toward resources that can alleviate their burden, caregivers can avoid decline in the emotional and physical functioning or even improve their current condition.

The findings also emphasize that the government plays an important role in supporting financially older caregivers. Even though the financial support was relatively small, in opinions of many participants, it helped them to feel appreciated and helped to cope with financial strains. Although the effectiveness of the intervention requires additional validation, the results are encouraging as they suggest that even a small financial aid combined with educational training can decrease caregiver burden. Reduced caregiver burden may, in turn, lead to lower rates of institutionalization and decrease the economic costs related to the growing number of individuals with AD.

## Funding

This work was supported by the grant of National Science Centre (grant no. 2017/01/X/HS6/02016). The funding bodies had no role in study design, data collection, or analysis.

## Conflict of Interest

None reported.
